# Supplementary material for: Structural basis for human mitochondrial tRNA maturation
Source: Nat Commun. 2024 Jun 1;15:4683. doi: 10.1038/s41467-024-49132-0 (PMC11144196; doi:10.1038/s41467-024-49132-0)
Supplement: Supplementary file 1 — Supplementary Information [file 41467_2024_49132_MOESM1_ESM.pdf]

Supplementary Information for:

Structural basis for human mitochondrial tRNA maturation

Vincent Meynier[1], Steven W. Hardwick[2], Marjorie Catala[1], Johann J. Roske [2], Stephanie Oerum[1], Dimitri Y. Chirgadze[2], Pierre Barraud[1], Wyatt W. Yue[3,4], Ben F. Luisi[2] & Carine Tisné[1]*

[1]Expression Génétique Microbienne, Université Paris Cité, CNRS, Institut de Biologie Physico-Chimique (IBPC), 75005 Paris, France.
[2]Department of Biochemistry, University of Cambridge, Tennis Court Road, Cambridge CB2 1GA, U.K.
[3]Centre for Medicines Discovery, Nuffield Department of Clinical Medicine, University of Oxford, Oxford OX3 7DQ, UK
[4]Present address: Biosciences Institute, Newcastle University, Newcastle upon Tyne, NE2 4HH, UK

The file includes Supplementary Table 1 and Supplementary Fig. 1-14.

Supplementary Table 1. Cryo-EM data collection and refinement statistics

Structure	Complex 1<br>TRMT10C/SDR5C1<br>pre-tRNA^{Ile}	Complex 2<br>TRMT10C/SDR5C1<br>pre-tRNA$^{His-Ser}$<br>PRORP	Complex 3<br>TRMT10C/SDR5C1<br>pre-tRNA$^{His(0,Ser)}$<br>ELAC2	Complex 4<br>TRMT10C/SDR5C1<br>pre-tRNA^{Ile}<br>TRNT1
PDB code	8CBO	8CBK	8CBL	8CBM
EMDB code	EMD-16547	EMD-16544	EMD-16543	EMD-16545
**Data collection**				
EM equipment	Titan Krios	Titan Krios	Titan Krios	Titan Krios
Voltage (kV)	300	300	300	300
Detector	Gatan K3	Gatan K3	Gatan K3	Gatan K3
Mode	Counting, super resolution	Counting, super resolution	Counting super resolution	Counting, super resolution
Nominal magnification	105,000x	130,000x	130,000x	130,000x
Pixel size (Å per pixel area)	0.723	0.652	0.652	0.652
Electron dose, total (e-/ Å^2)	40.10	48.52	48.90	42,20
Defocus range (μm)	-0.9 to -2.5	-0.9 to -2.4	-0.9 to -2.4	-0.8 to -2.2
Exposure (s)	3.0	1.34	1.34	1.24
Frames	40	150	50	46
Number of micrographs	6,474	7,573	10,927	9,644
**Processing**				
Software	Relion 3.1.0	WARP<br>CryoSPARC v3.1	WARP<br>Cryosparc v3.1	WARP<br>CryoSparc v4.0
Initial number of particles	1,536,128	1,789,726	2,049,642	2,420,733
Final number of particles	141,653	81,396	74,166	25,554
Symmetry imposed	C1	C1	C1	C1
Map resolution, $FSC_{0.143}$ (Å)	3.20	2.76 (Map 1)<br>2.74 (Map 2)<br>4.50 (Map 3)	2.79 (Map 1)<br>2.47 (Map 2)<br>4.05 (Map 3)	3.14 (Map 1)<br>4.50 (Map 2)
Map resolution range (Å)	2.6-6.5	2.5-6.8 (Map 1)<br>2.5-6.5 (Map 2)<br>4.0-7.9 (Map 3)	2.5-6.8 (Map 1)<br>2.5-6.8 (Map 2)<br>3.6-8.8 (Map 3)	2.9-7.2 (Map 1)<br>4.0-7.2 (Map 2)
Map-sharpening B-factor (Å^2)	-133.7	-70.7 (Map 1)<br>-68.5 (Map 2)<br>-246.1 (Map 3)	-69.9 (Map 1)<br>-63.8 (Map 2)<br>-191.3 (Map 3)	-60.7 (Map 1)<br>-183.4 (Map 2)
**Model composition**				
Non-hydrogen atoms	10626	16223	17251	14576
Protein residues	1232	1834	2007	1701
RNA bases	58	93	86	66
Ligand	5	7	6	6
**Refinement**				
Initial model used (PDB code)	7ONU, 5NFJ, Complex 4	7ONU, 5NFJ AF-Q7L0Y3, AF-O15091, Complex 3	7ONU, 5NFJ, AF-Q7L0Y3, AF-Q9BQ52, Complex 2	7ONU, 5NFJ, AF-Q7L0Y3, AF-Q96Q11, Complex 1
Software	Phenix-1.20.1-4487	Phenix-1.20.1-4487	Phenix-1.20.1-4487	Phenix-1.20.1-4487
Correlation coefficient, masked	0.81	0.84	0.75	0.83
**Model validation**				

MolProbity score	1.58	1.61	1.57	1,66
All-atom clash score	5.37	6.17	5.97	7.65
Poor rotamers	0.00	0.00	0.00	0.00
Ramachandran statistics (%):				
Favoured (overall)	95.74	95.99	96.34	96.38
Allowed (overall)	4.26	4.01	3.66	3.62
Outliers (overall)	0.00	0.00	0.00	0.00
RMS deviations:				
Bond length (Å)	0.003	0.002	0.002	0.003
Bond angle (°)	0.480	0.476	0.486	0.446

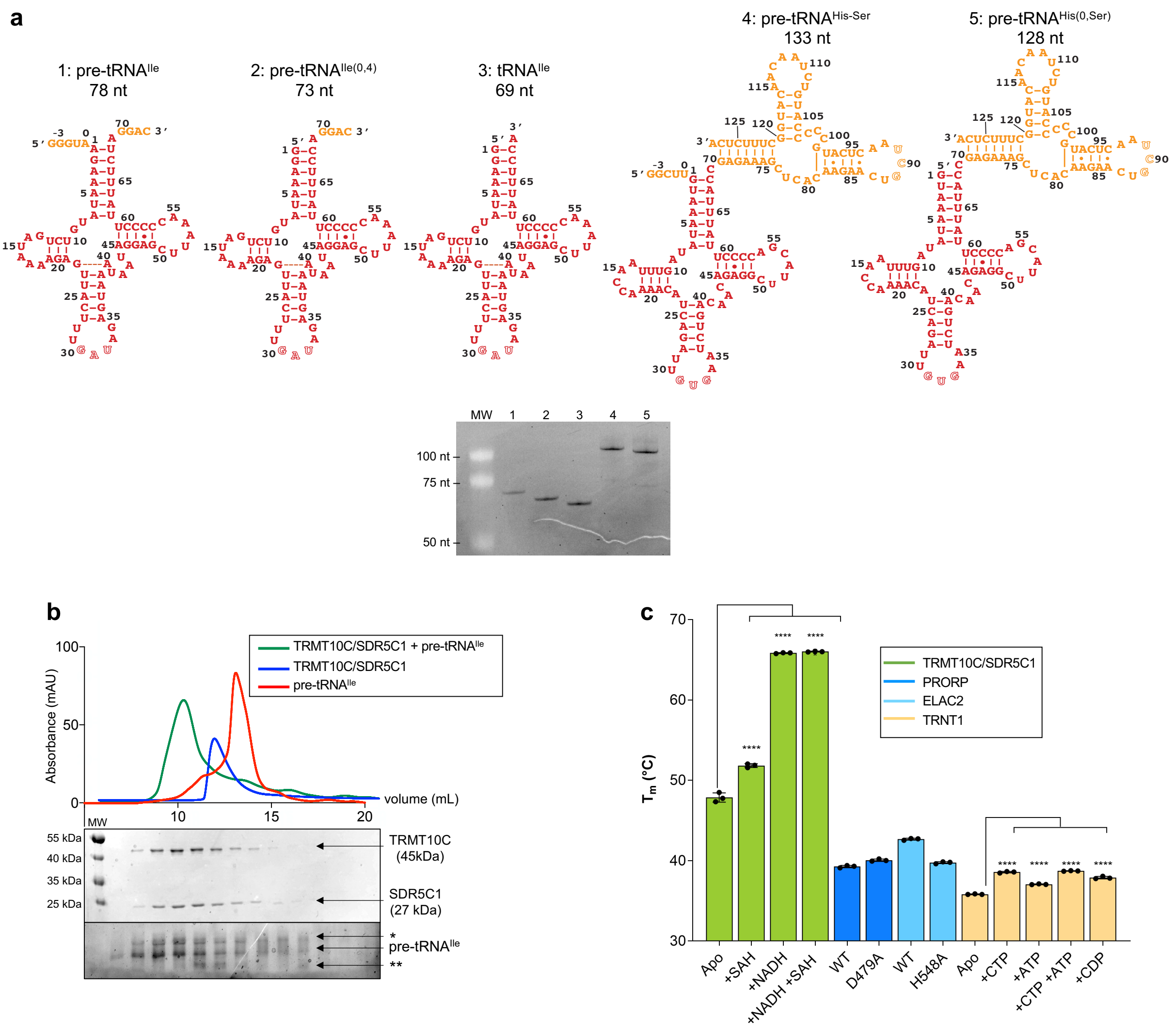

Supplementary Fig. 1 | Biochemical characterization of TRMT10C/SDR5C1/tRNA^{Ile} complex and stability of the maturation enzymes in presence of their cofactors or analogues. **a** Secondary structure of pre-tRNA used in this study, the 5'-leader and 3'-trailer are indicated in orange, tRNA^{Ser}(AGY) is the 3'-trailer of tRNA^{His}, the nucleotide numbering (in black) is that used in the structure coordinates deposited in the PDB. 16% urea gel analysis of the tRNA samples used in this study. **b** Analytical size exclusion chromatography (Superdex 200 increase 10/300 GL) of TRMT10C/SDR5C1 in presence of pre-tRNA^{Ile} which confirms that TRMT10C/SDR5C1 forms a stable complex with pre-tRNA^{Ile}, 14% SDS-PAGE gel analysis of the fractions stained first with ethidium bromide to observe the pre-tRNA and then with Coomassie blue to observe the proteins. The high (*) and low (**) molecular weight bands are present in the pre-tRNA sample alone and probably correspond to species that are not properly folded. **c** Melting temperatures (Tm, in °C) measured from DSF experiments of TRMT10C/SDR5C1 (in green), PRORP and PRORP$_{D479A}$ (in dark blue), ELAC2 and ELAC2$_{H548A}$ (in cyan) and TRNT1 (in beige) with no ligand (Apo) or in the presence of cofactors or analogues. Data were analysed by one-way ANOVA using a Dunnett's multiple comparison test, n = 3 technical replicates, **** for P < 0.0001. Source data are provided as a Source Data file.

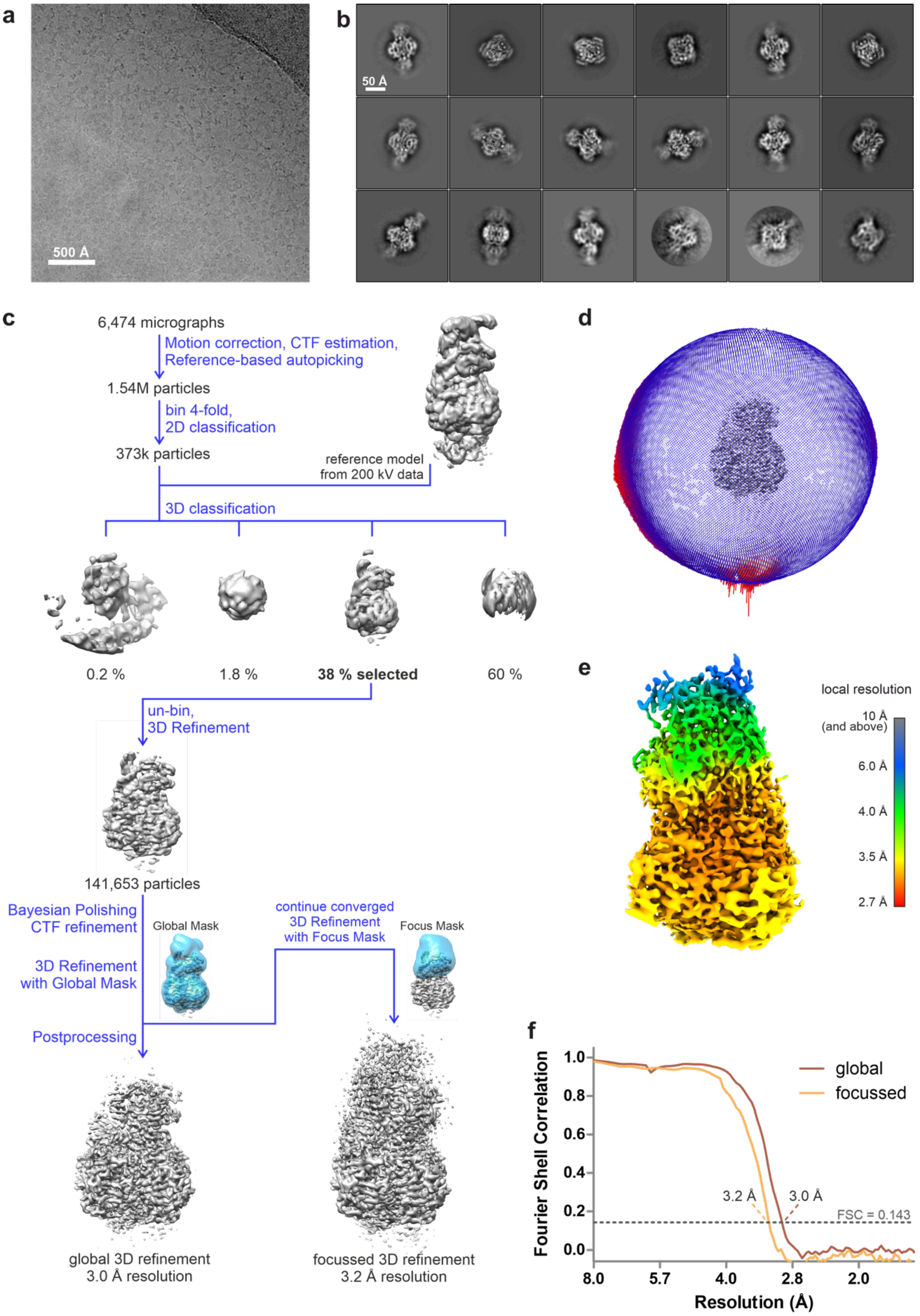

Supplementary Fig. 2 | Cryo-EM analysis of the TRMT10C/SDR5C1/pre-tRNA^{Ile} complex. a Example micrograph after Motion Correction and CTF estimation. Scale bar, 50 nm. **b** Representative 2D class averages generated in Relion using the final particle set. Scale bar, 5 nm. **c** Image processing workflow. **d**,3D representation of angular distributions of the particles used for the final map. **e** Refined 3D map coloured by local resolution calculated in Relion. **f** Fourier Shell Correlation (FSC) plots for the two final refinements using global or focus masks (shown in (c)), respectively. Resolution is indicated at the FSC=0.143 cut-off.

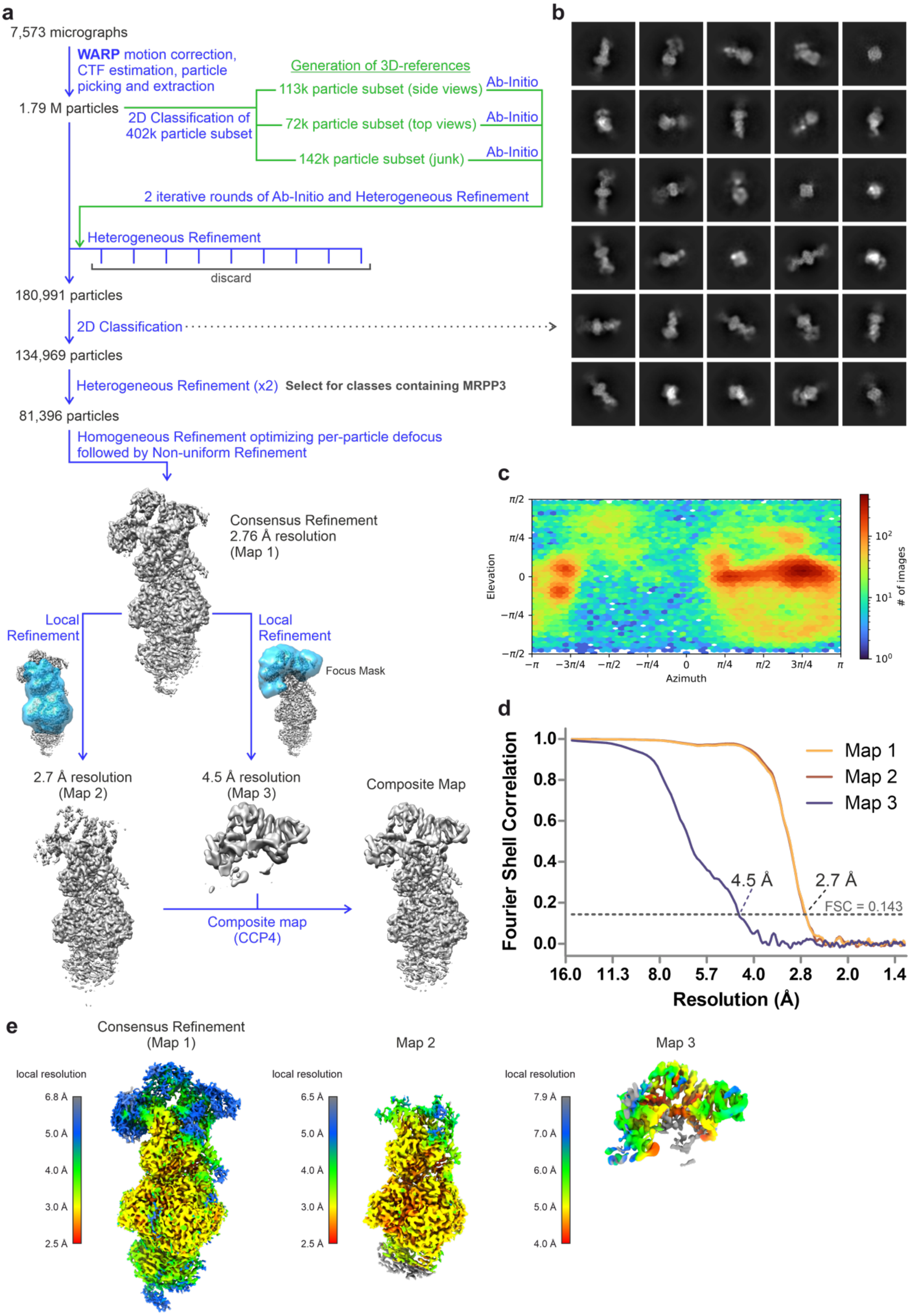

Supplementary Fig. 3 | Cryo-EM analysis of the TRMT10C/SDR5C1/pre-tRNA$^{His\text{-}Ser}$/PRORP complex. a Image processing workflow. **b** Representative 2D class averages generated in cryoSPARC. **c** Angular distribution plot of the final particle set after consensus refinement. **d** Fourier Shell Correlation (FSC) plots for the three final refinements shown in (a), Maps 1-3, respectively. Resolution is indicated at the FSC=0.143 cut-off. **e** Maps 1-3 from the three final refinements (a), coloured by local resolution calculated in CryoSPARC.

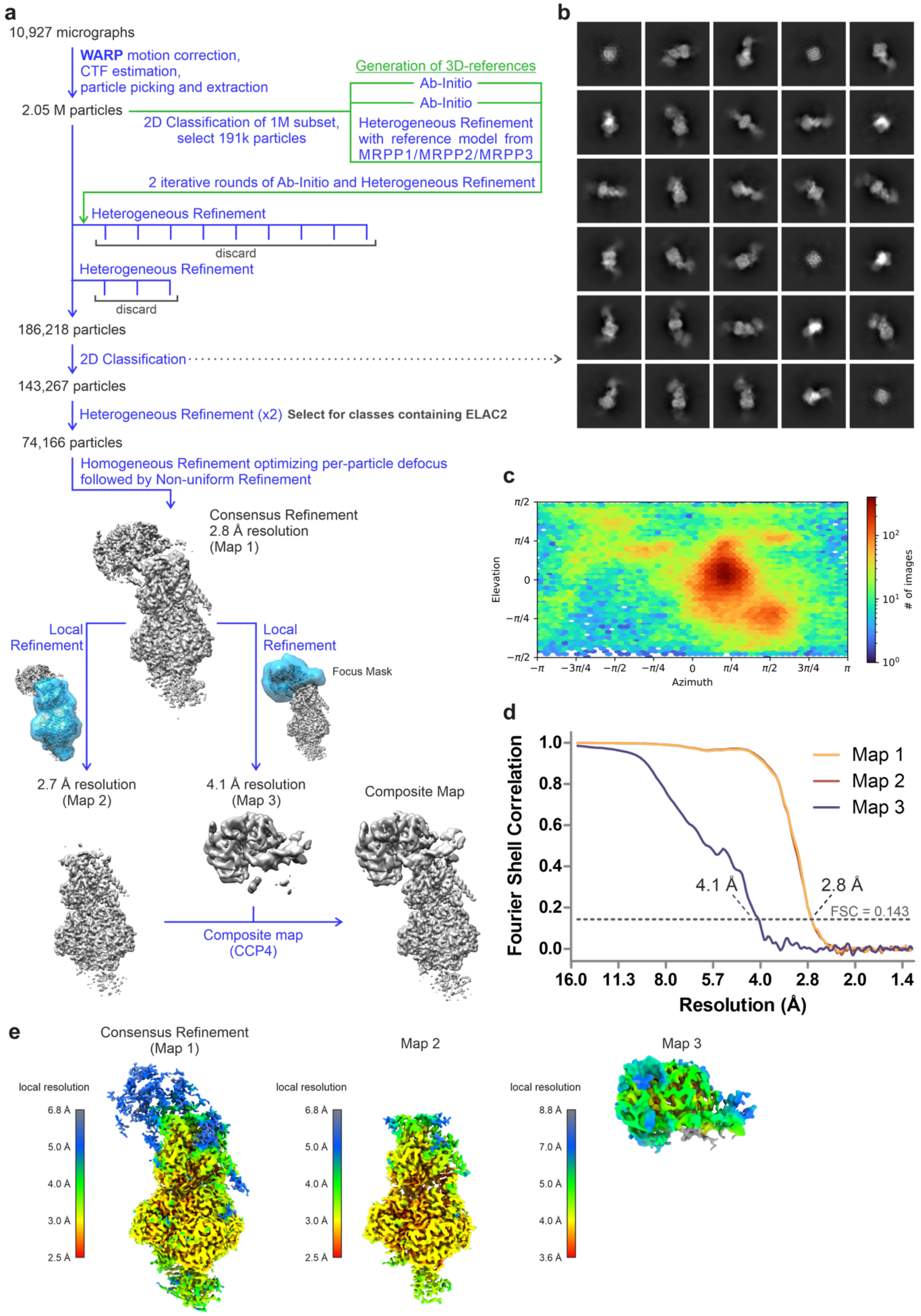

Supplementary Fig. 4 | Cryo-EM analysis of the TRMT10C/SDR5C1/pre-tRNA$^{His(0,Ser)}$/ELAC2 complex. a Image processing workflow. **b** Representative 2D class averages generated in cryoSPARC. **c** Angular distribution plot of the final particle set after consensus refinement. **d** Fourier Shell Correlation (FSC) plots for the three final refinements shown in (a), Maps 1-3, respectively. Resolution is indicated at the FSC=0.143 cut-off. **e** Maps 1-3 from the three final refinements (a), coloured by local resolution calculated in CryoSPARC.

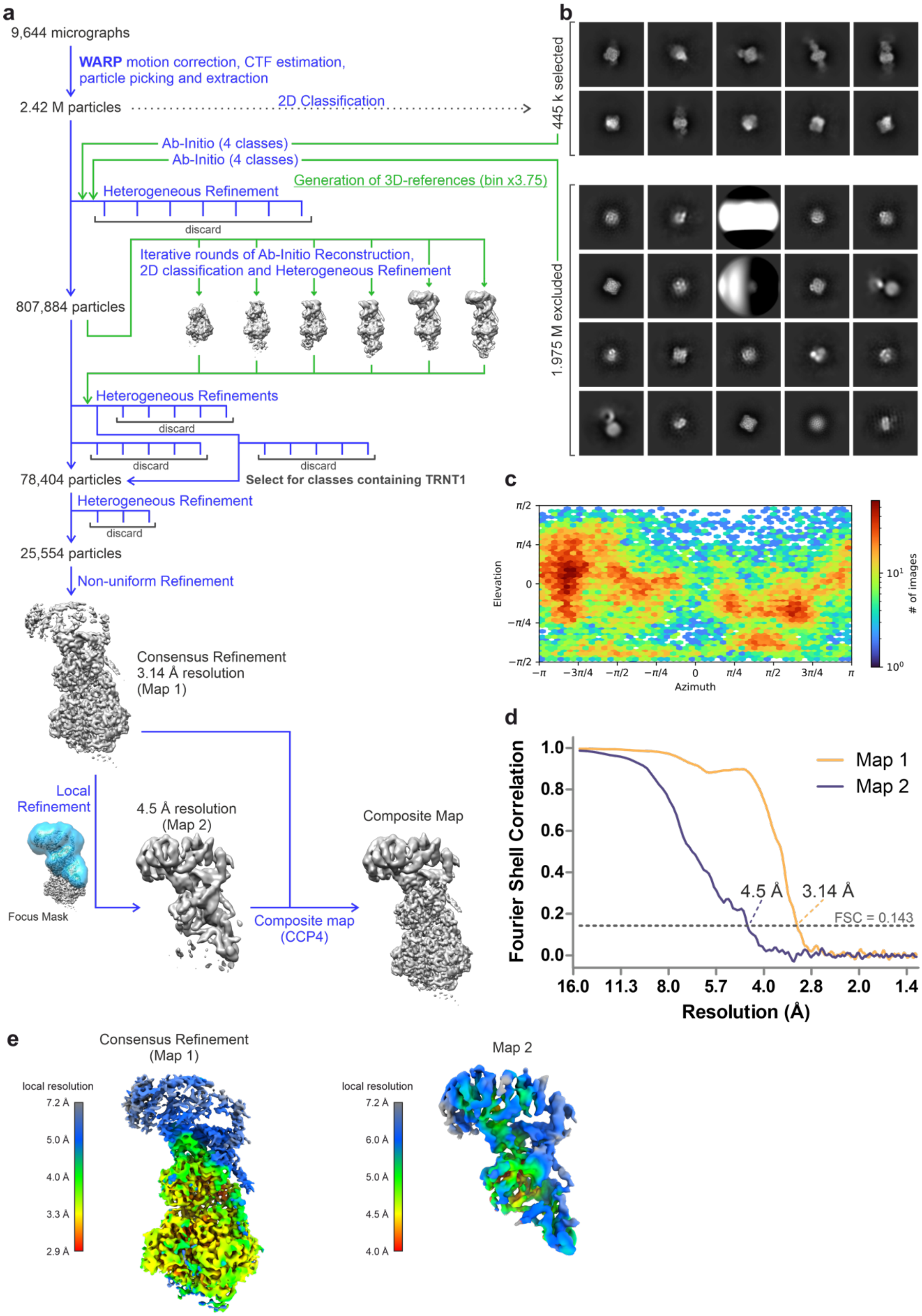

Supplementary Fig. 5 | Cryo-EM analysis of the TRMT10C/SDR5C1/pre-tRNA^{Ile}/TRNT1 complex. a Image processing workflow. **b** Representative 2D class averages generated in cryoSPARC. **c,** Angular distribution plot of the final particle set after consensus refinement. **d** Fourier Shell Correlation (FSC) plots for the two final (consensus and local) refinements shown in (a). Resolution is indicated at the FSC=0.143 cut-off. **e** Maps 1 and 2 from the final consensus and local refinements (a), coloured by local resolution calculated in CryoSPARC.

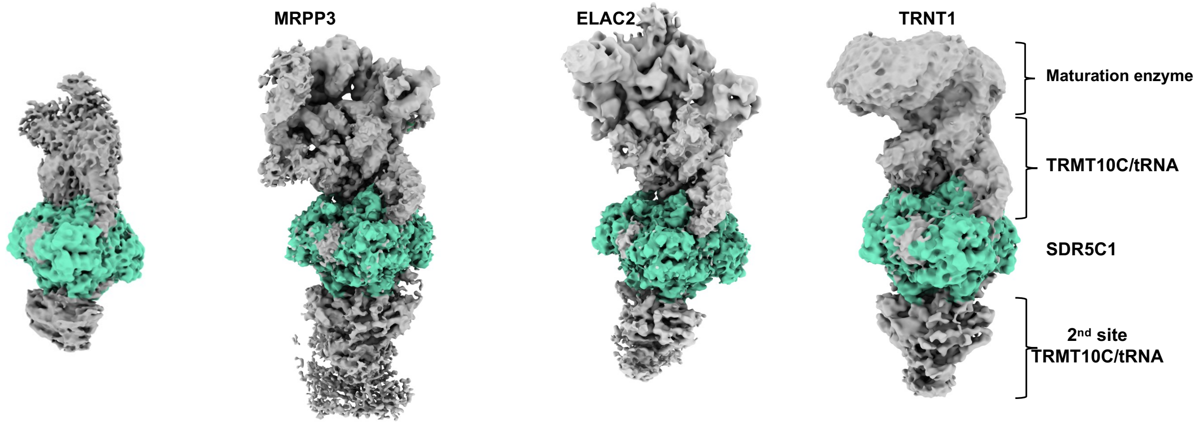

Supplementary Fig. 6 | cryo-EM density maps of the human mt tRNA maturation complexes. Consensus maps showing the second site of binding of the TRMT10C/tRNA complex on SDR5C1 from left to right: TRMT10C/SDR5C1/pre-tRNA^{Ile}, TRMT10C/SDR5C1/pre-tRNA$^{His-Ser}$/PRORP$_{D479A}$, TRMT10C/SDR5C1/pre-tRNA$^{His(0,Ser)}$/ELAC2$_{H548A}$, TRMT10C/SDR5C1/tRNA^{Ile}/TRNT1/CDP.

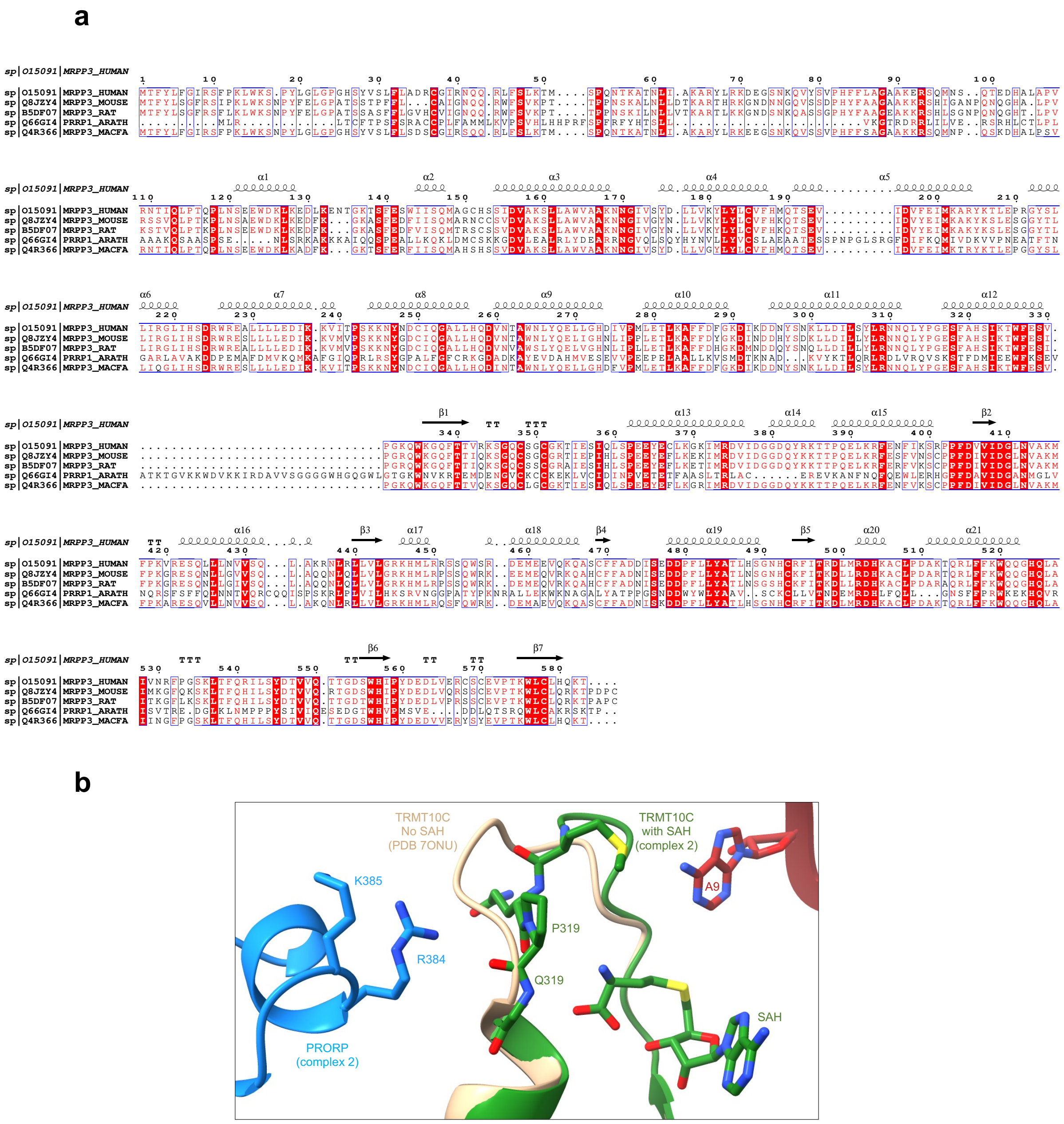

Supplementary Fig. 7 | PRORP sequence conservation and PRORP interaction with TRMT10C active site. **a** Multiple sequence alignment of a representative set of PRORP sequences with colour coding from ESPript[1]. The secondary structure of the human PRORP is shown above the alignment and the sequences are numbered according to human PRORP. Abbreviations for sequences are as follows: HUMAN: *Homo Sapiens* (Uniprot O15091), Mouse: *Mus musculus* (Uniprot Q8JZY4), RAT: *Rattus norvegicus* (Uniprot B5DF07), ARATH: *Arabidopsis thaliana* (Uniprot Q66GI4), MACFA: *Macaca fascicularis* (Uniprot Q4R366). **b** Movement of the SAM-binding loop in the MTase domain of TRMT10C upon binding of SAH, TRMT10C in complex 2 in green and PRORP in complex 2 in blue, the structure with tRNA and without SAH is PDB 7ONU (in beige).

a

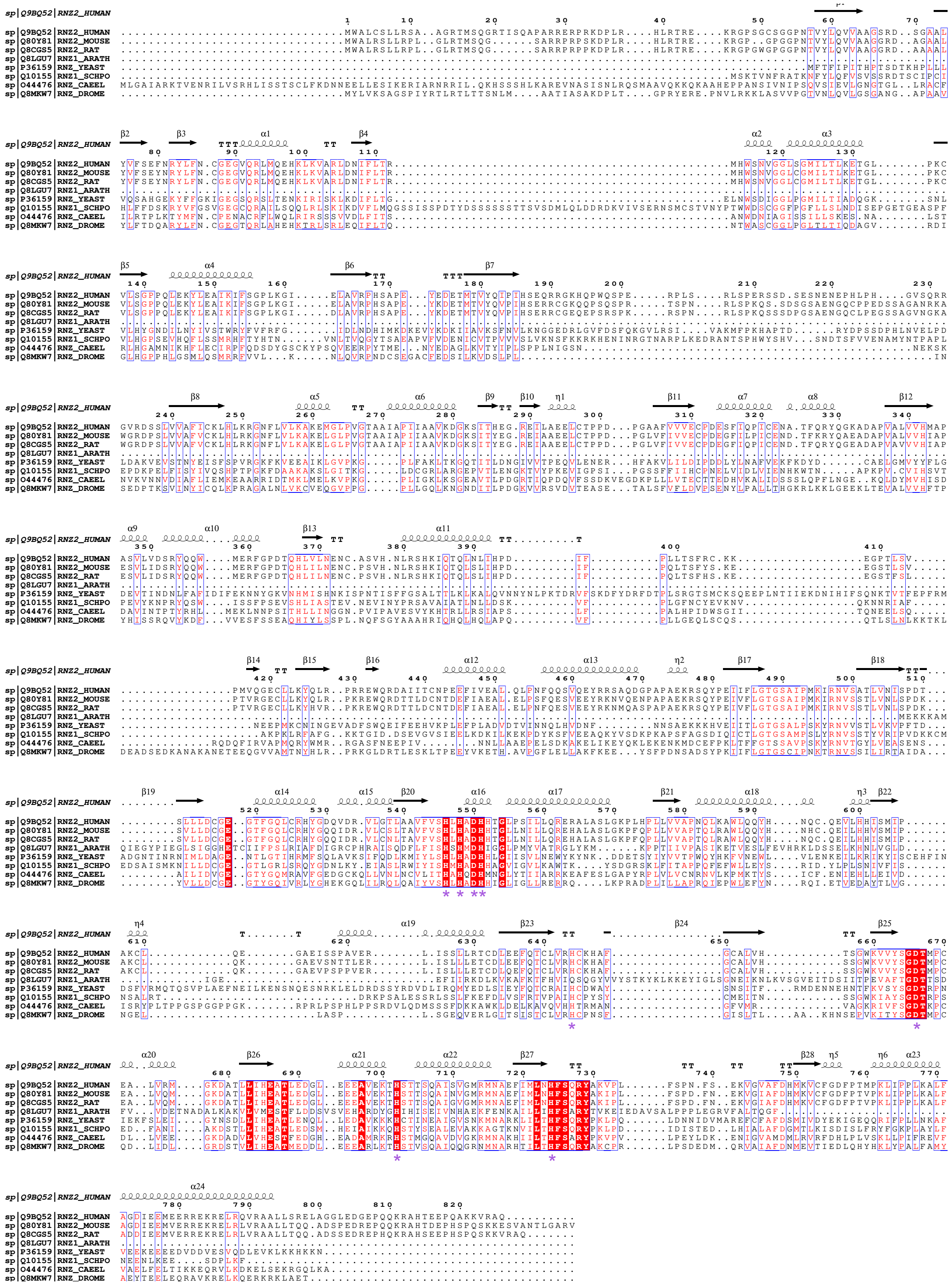
sp|Q9BQ52|RNZ2_HUMAN
sp|Q80Y81|RNZ2_MOUSE
sp|Q8CGS5|RNZ2_RAT
sp|Q8LGU7|RNZ1_ARATH
sp|P36159|RNZ_YEAST
sp|Q10155|RNZ1_SCHPO
sp|O44476|RNZ_CAEEL
sp|Q8MKW7|RNZ_DROME

b

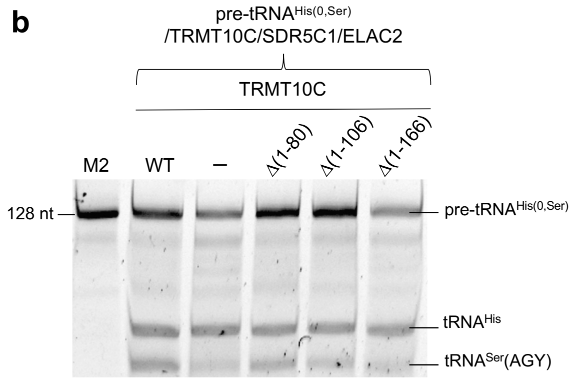

Supplementary Fig. 8 | Conserved residues in ELAC2 and ELAC2 cleavage assays on a pre-tRNA bound in TRMT10C deletion mutants/SDR5C1 complex. a Multiple sequence alignment of a representative set of ELAC2 sequences with colour coding from ESPript[1]. The secondary structure of the human ELAC2 is shown above the alignment and the sequences are numbered according to ELAC2. Abbreviations for sequences are as follows: HUMAN: *Homo sapiens* (Uniprot Q9BQ52), MOUSE: *Mus musculus* (Uniprot Q80Y81), RAT: *Rattus norvegicus* (Uniprot Q8CGS5), ARATH: *Arabidopsis thaliana* (Uniprot Q8LGU7), YEAST: *Saccharomyces cerevisiae* (Uniprot P36159), SCHPO: *Schizosaccharomyces pombe* (Uniprot Q10155), CAEEL: *Caenorhabditis elegans* (Uniprot O44476) and DROME: *Drosophila melanogaster* (Uniprot Q8MKW7). The stars in purple indicate the residues involved in Mg^{2+} coordination in the catalytic site of ELAC2. **b** Cleavage assays of ELAC2 on pre-$tRNA^{His(0,Ser)}$ using TRMT10C deletion mutants in complex with SDR5C1. Marker M2 is pre-$tRNA^{His(0,Ser)}$ (128 nt). WT: TRMT10C/SDR5C1/ELAC2/pre-$tRNA^{His(0,Ser)}$, (-) : ELAC2/pre-$tRNA^{His(0,Ser)}$. The gel is representative of three experiments, uncropped gel is in Source Data file.

a

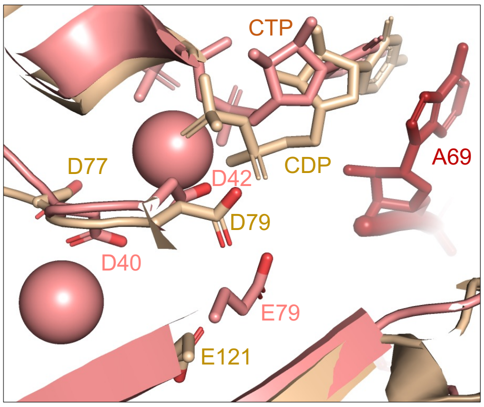
CTP
D77
CDP
A69
D42
D79
D40
E79
E121

b

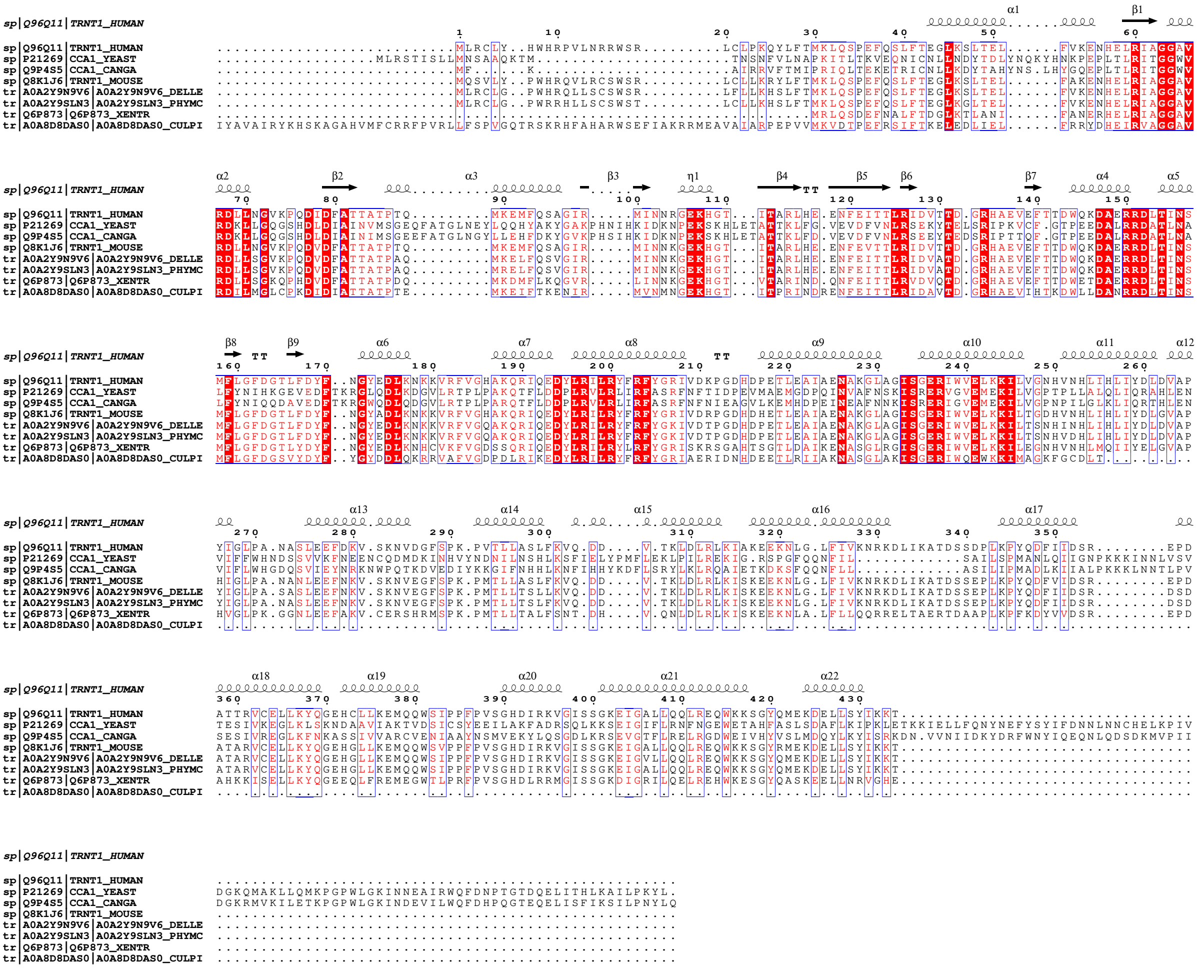
sp|Q96Q11|TRNT1_HUMAN
sp|Q96Q11|TRNT1_HUMAN
sp|P21269|CCA1_YEAST
sp|Q9P4S5|CCA1_CANGA
sp|Q8K1J6|TRNT1_MOUSE
tr|A0A2Y9N9V6|A0A2Y9N9V6_DELLE
tr|A0A2Y9SLN3|A0A2Y9SLN3_PHYMC
tr|Q6P873|Q6P873_XENTR
tr|A0A8D8DAS0|A0A8D8DAS0_CULPI

c

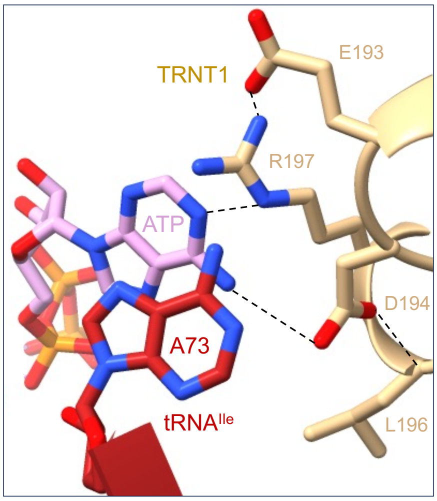

Supplementary Fig. 9 | TRNT1 metal-binding and sequence conservation. a Superimposition of the TRNT1 active site (in beige) with the crystal structure of *B. stearothermophilus* CCA-adding enzyme in complex with CTP and Mg^{2+} ions (in light magenta, PDB 1MIY). **b** Multiple sequence alignment of a representative set of TRNT1 sequences with colour coding from ESPript[1]. The secondary structure of the human TRNT1 is shown above the alignment and the sequences are numbered according to human TRNT1. Abbreviations for sequences are as follows: HUMAN: *Homo Sapiens* (Uniprot Q96Q11), YEAST: *Saccharomyces cerevisiae* (Uniprot P21269), CANGA: *Candida glabrata* (Uniprot Q9P4S5), MOUSE: *Mus musculus* (Uniprot Q8K1J6), DELLE: *Delphinapterus leucas* (Uniprot A0A2Y9N9V6), PHYMC: *Physeter macrocephalus* (Uniprot A0A2Y9SLN3), XENTR: *Xenopus tropicalis* (Uniprot Q6P873) and CULPI: *Culex pipiens* (Uniprot A0A8D8DAS0). **c** Base-specific interactions between TRNT1 and ATP (ATP from PDB 1MIW).

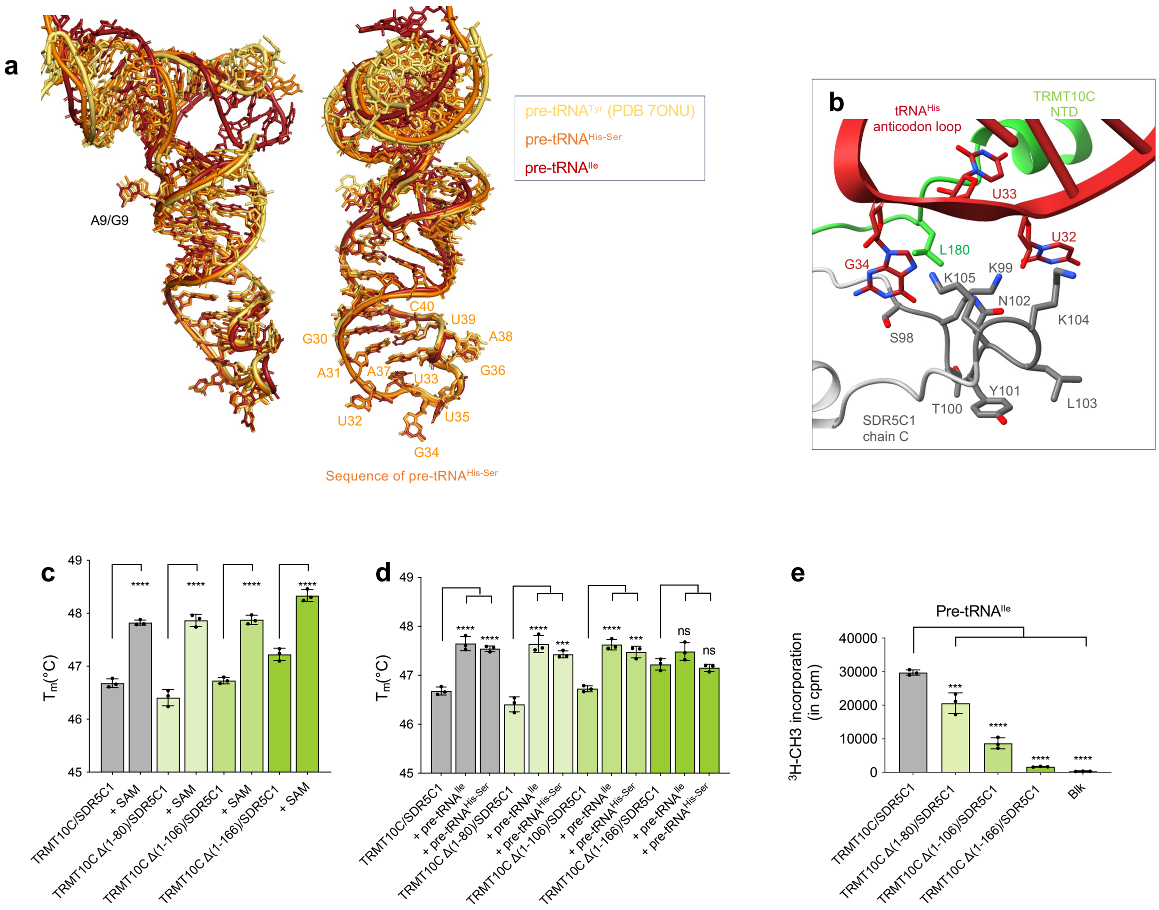

Supplementary Fig. 10 | mt tRNA interactions with TRMT10C. a Superimposition of pre-tRNA$^{His\text{-}Ser}$ (in orange), pre-tRNA^{Ile} (in red) and pre-tRNA^{Tyr} (PDB 7ONU, in yellow), tRNA^{Ser} is not shown for the sake of clarity. **b** Interaction between the anticodon loop of tRNA^{His} and SDR5C1. **c** Melting temperatures (Tm, in °C) of TRMT10C deletion mutants in presence or in absence of SAM, TRMT10C/SDR5C1 WT (in grey), TRMT10C Δ(1-80)/SDR5C1 in light green, TRMT10C Δ(1-106)/SDR5C1 in green and TRMT10C Δ(1-166)/SDR5C1 in lime, measured from DSF experiments. Data were analysed by one-way ANOVA using a F test statistic, n=3 technical replicates, **** is for P-value < 0.0001. **d** Melting temperatures (Tm, in °C) of TRMT10C deletion mutants in presence of pre-tRNA^{Ile} or pre-tRNA$^{His\text{-}Ser}$ measured from DSF experiments, color as in b. Data were analysed by one-way ANOVA using a F test statistic, n=3 technical replicates, P-values are indicated as follow: ns for 0.0846 (TRMT10C Δ(1-166)/SDR5C1) or 0.7492 (Blk), *** for P = 0.0002 and **** for P < 0.0001. **e** MTase assays of TRMT10C deletion mutants on pre-tRNA^{Ile}, Blk: assay without pre-tRNA, color as in b. Data were analysed by one-way ANOVA using a Dunnett's multiple comparison test, n=3 technical replicates, P-values are indicated as follows: *** for P = 0.001 and ****for P < 0.0001. Source data are provided as a Source Data file.

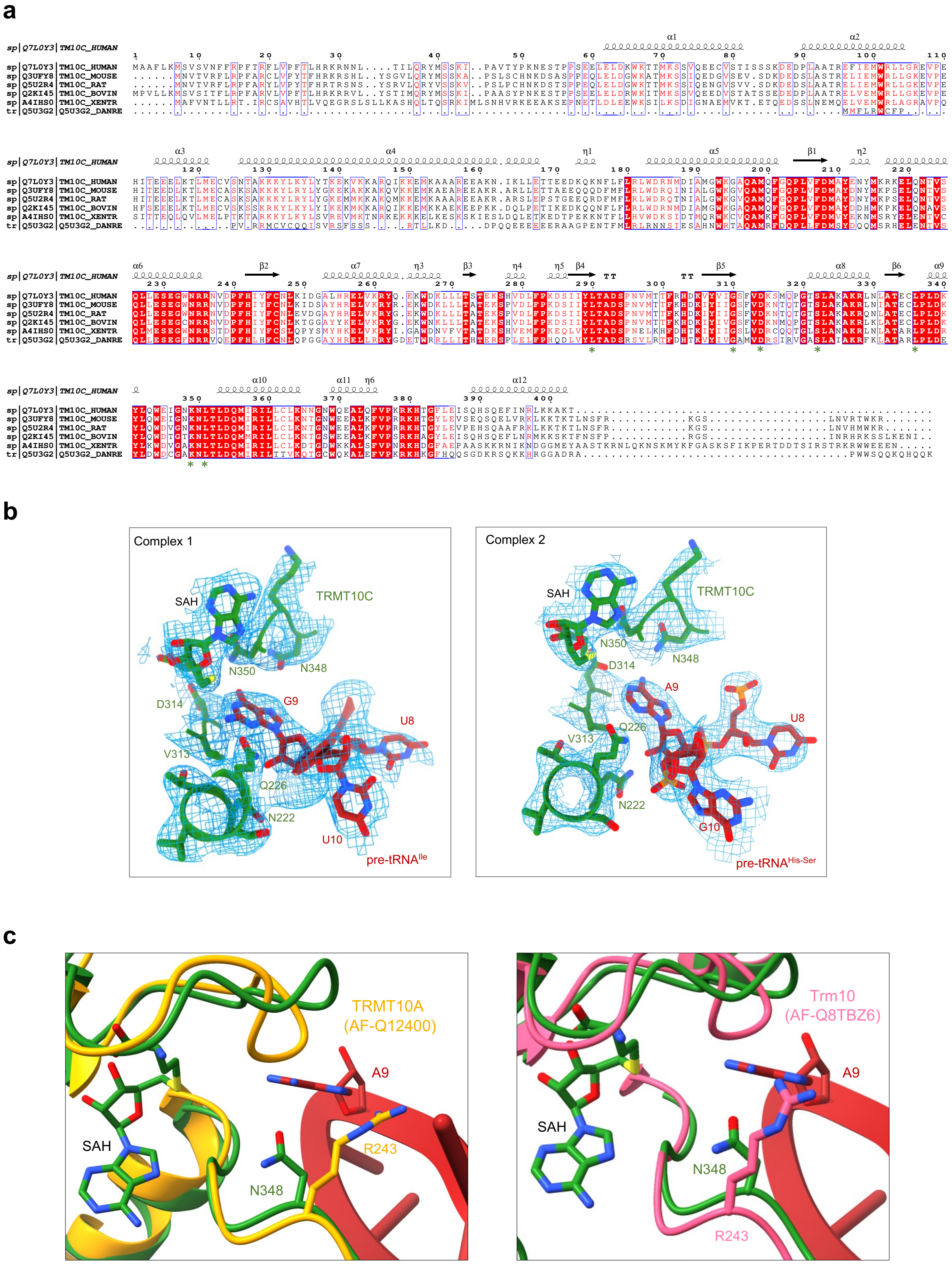

Supplementary Fig. 11 | TRMT10C sequence conservation and active site organization. a Multiple sequence alignment of a representative set of TRMT10C sequences from eukaryotes with colour coding from ESPript[1]. The secondary structure of the human TRMT10C is shown above the alignment and the sequences are numbered according to human TRMT10C. Abbreviations for sequences are as follows: HUMAN: *Homo sapiens* (Q7L0Y3), MOUSE: *Mus musculus* (Uniprot Q3UFY8), RAT: *Rattus norvegicus* (Uniprot Q5U2R4), BOVIN: *Bos taurus* (Uniprot Q2KI45), XENTR: *Xenopus tropicalis* (Uniprot A4IHS0) and DANRE: *Danio rerio* (Uniprot Q5U3G2). Green stars indicate TRMT10C residues involved in SAH binding. **b** Model-to-map fit for G9 and SAH in complex 1 and A9 and SAH in complex 2. **c** Comparison of the active sites of TRMT10C (m^1A9/m^1G9 activity) with human TRMT10A (left, AF-Q12400) and *S. cerevisae* Trm10 (right, AF-Q8TBZ6), both with m^1G9 MTase activity.

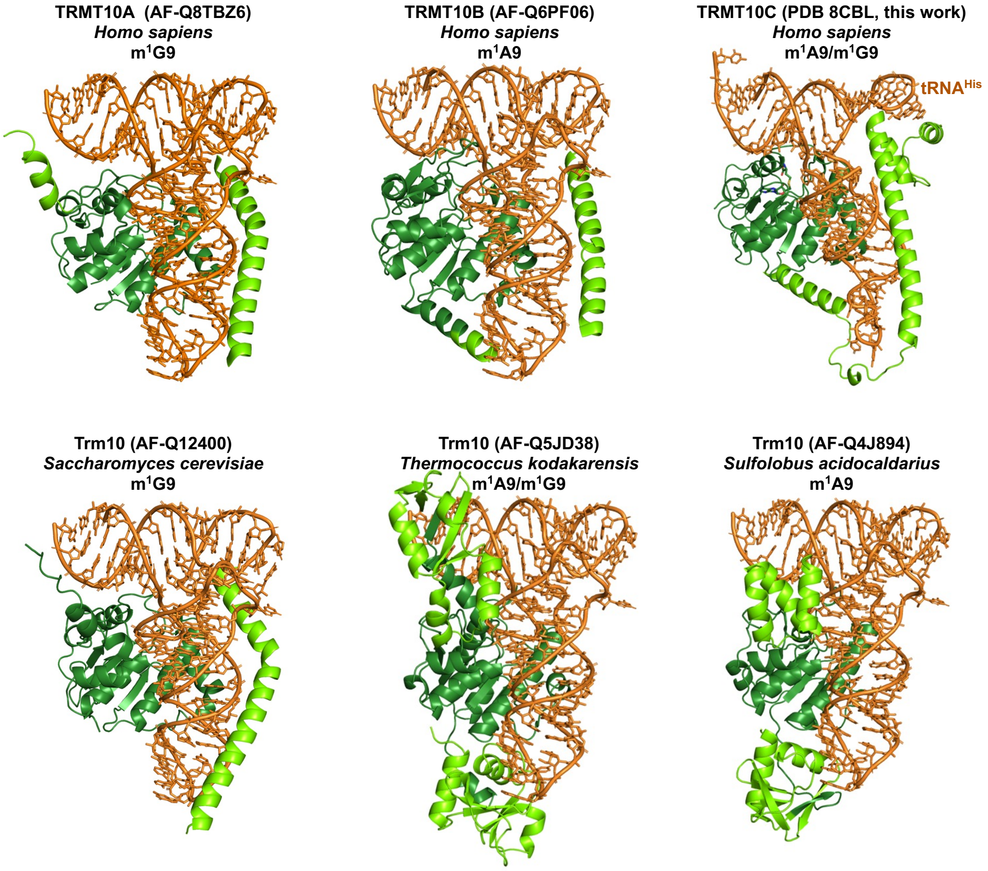

Supplementary Fig. 12 | Models of Trm10-family enzyme/tRNA complexes, highlighting the different strategies to bind tRNA. The models were generated using the structure of TRMT10C bound to tRNA$^{His\text{-}Ser}$ (PDB 8CBL, this work), tRNA^{Phe} (PDB 6LVR) and Alphafold models of the Trm10-family enzymes.

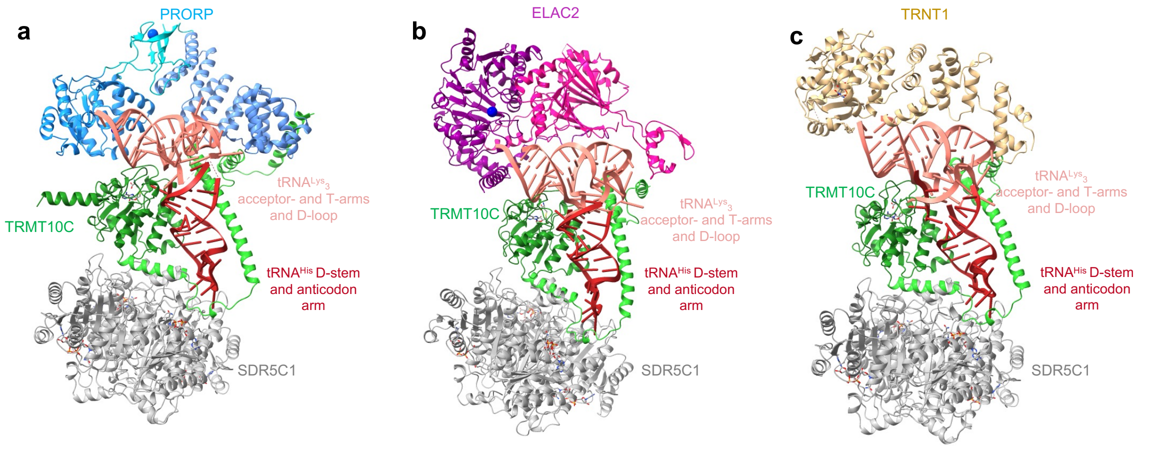

Supplementary Fig. 13 | Models of tRNA maturation complexes for a tRNA with a stable elbow.
The models were generated using the structure of mitochondrial tRNA maturation complexes of complex 2, 3 and 4 (Fig. 1) with the human $tRNA^{Lys}{}_3$ acceptor- and T-arms plus the D-loop (in pink) aligned on the $tRNA^{His}$ structure (in red).

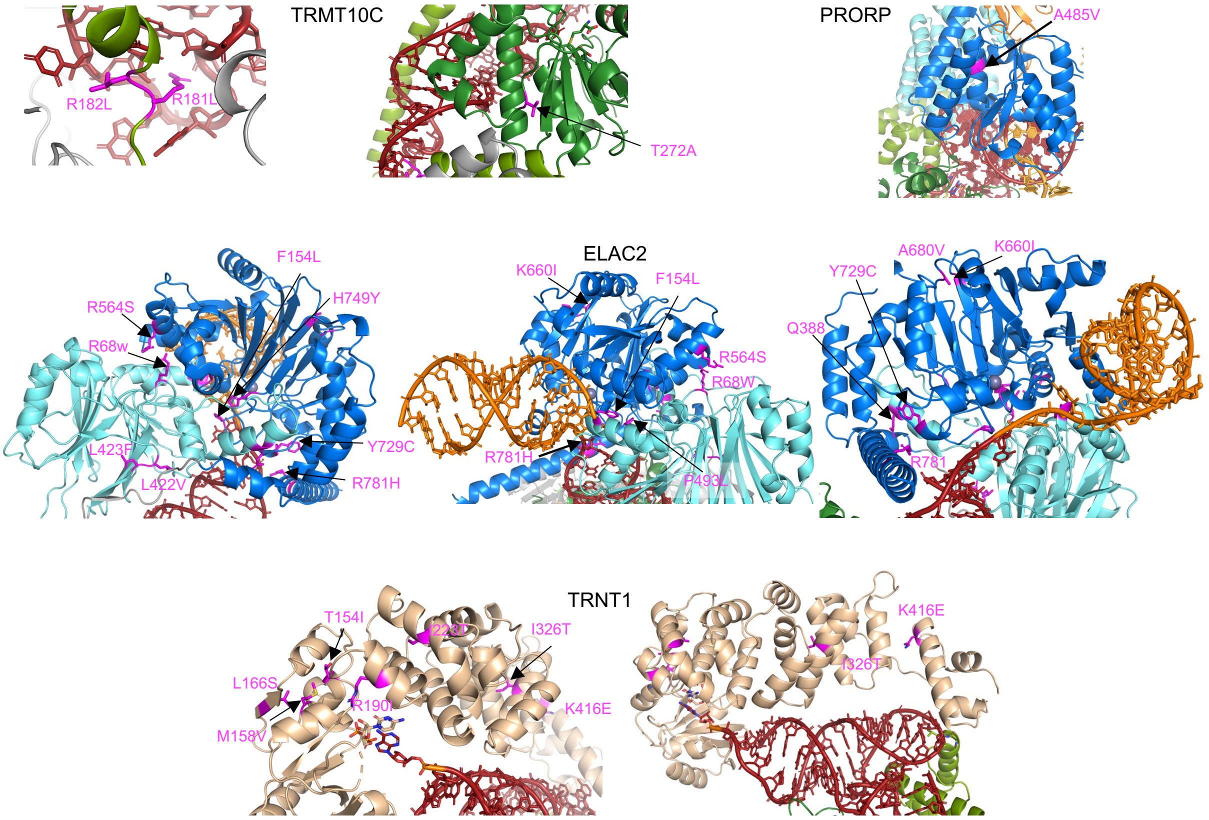

Supplementary Fig. 14 | Structural mapping of disease mutations on the enzymes involved in human mt pre-tRNA maturation. Disease mutations are indicated in pink.

References

1. Robert, X. & Gouet, P. Deciphering key features in protein structures with the new ENDscript server. *Nucleic Acids Res* **42**, W320-324 (2014).
