# Supplementary material for: Structural basis for human mitochondrial tRNA maturation
Source: Nat Commun. 2024 Jun 1;15:4683. doi: 10.1038/s41467-024-49132-0 (PMC11144196; doi:10.1038/s41467-024-49132-0)
Supplement: Supplementary file 3 — Description of Additional Supplementary Information [file 41467_2024_49132_MOESM3_ESM.pdf]

Description of Additional Supplementary Information

Supplementary Movie 1: Movie summarizing human mitochondrial pre-tRNA maturation. The video shows our model of the pre-tRNA binding by TRMT10C/SDR5C1 subcomplex and PRORP recruitment to form the mitochondrial RNase P complex, the exchange of PRORP with ELAC2 to form the RNase Z complex and the exchange with TRNT1 to complete tRNA maturation at the 3'-end. Protein subunits are shown in cartoon, pre-tRNA is shown as ribbon and sticks, subunit coloring is the same as in Fig. 1.
